# Extracellular Tau and Its Potential Role in the Propagation of Tau Pathology

**DOI:** 10.3389/fnins.2017.00667

**Published:** 2017-11-29

**Authors:** Kaoru Yamada

**Affiliations:** Department of Neuropathology, Graduate School of Medicine, The University of Tokyo, Tokyo, Japan

**Keywords:** propagation, extracellular tau, tauopathies, Alzheimer's disease, unconventional protein secretion

## Abstract

The pathological aggregation of tau protein is a hallmark of a set of neurodegenerative diseases collectively referred to as tauopathies. Tau aggregates independently in each neuron, but this aggregation can also occur in a non-cell autonomous manner in which aggregated tau is transmitted from one cell to another. Such trans-cellular propagation is initiated by the uptake of extracellular tau, which then seeds soluble tau in the recipient cells to spread the tau pathology. Accumulating evidence has demonstrated that tau is not only present in the cytoplasm of neurons but also actively released into the extracellular space. This finding has led to the idea that extracellular tau could be a novel therapeutic target to halt the propagation of tau pathology. From this perspective, the present review article focuses on recent advances in understanding the mechanisms regulating the levels of extracellular tau and discusses the role of such mechanisms in the propagation of tau pathology.

## Introduction

The abnormal accumulation of tau protein as neurofibrillary tangles is the common pathological hallmark of a set of neurodegenerative diseases, including Alzheimer's disease (AD), that are collectively referred to as tauopathies. The deposition of neurofibrillary tangles highly correlates with cognitive decline in AD, suggesting that tau plays a critical role in neurodegeneration (Gómez-Isla et al., 1997). Tau is intrinsically highly soluble; however, in tauopathies, it self-assembles into fibrillar aggregates. This process involves a nucleation and elongation mechanism where tau first forms small intermediate oligomers that convert normally folded tau into misfolded aggregates by acting as aggregation nuclei or seeds (hereinafter referred to as “seed-competent tau”). Such nucleation-dependent aggregation was thought to occur only in a cell-autonomous manner, but compelling evidence now suggests that non-cell autonomous aggregation can also occur via seed-competent tau transmission from neurons to neurons via the extracellular space. The present review article discusses recent knowledge about the roles of extracellular tau including trans-cellular propagation, the possible mechanisms that regulate its extracellular levels, and the pathological implications of those mechanisms in tauopathies.

## Extracellular tau and its physiological/pathological roles

Tau is well-recognized for its role in assembling/stabilizing microtubules although tau single knockout mice do not show major phenotypic changes in neuronal microtubule stability because of a putative functional redundancy (Takei et al., 2000). Due to this interaction with microtubules, its localization has been long assumed to be restricted to the cytoplasm of neurons. However, accumulating evidence now demonstrates that tau is also physiologically present in the extracellular fluid. Tau is present in full-length (Karch et al., 2013) or truncated forms (Bright et al., 2015; Kanmert et al., 2015) not only in the culture media of cells over-expressing human tau (Chai et al., 2012), primary neurons (Karch et al., 2012), and iPSC derived neurons (Chai et al., 2012; Bright et al., 2015) but also in the brain interstitial fluid (ISF) and cerebrospinal fluid (CSF) of mice (Yamada et al., 2011) and human (Magnoni et al., 2012).

Although the physiological function of extracellular tau still remains unclear, extracellular tau currently receives considerable attention due to its role in trans-cellular propagation. Tau propagation was initially described in cell culture models where the extracellular application of tau fibrils enters the cells and robustly seeds aggregation of the soluble tau expressed in cells (Frost et al., 2009). Such a seeding effect was not observed when soluble monomeric tau was applied. Subsequent studies have shown that injecting tau fibrils into the brain also induces trans-neuronal propagation *in vivo* (Clavaguera et al., 2009; Iba et al., 2013). In cell culture studies, the transfer of conditioned media from cells with aggregates was sufficient to induce aggregation in the recipient cells (Kfoury et al., 2012; Wu et al., 2016), suggesting that seed-competent tau is released into the culture media. It remains uncertain that trans-cellular propagation also contributes to the progression of tau pathology in human (Walsh and Selkoe, 2016): nonetheless, seeding activity detected in human CSF using sensitive assays such as real-time quaking-induced conversion (RT-QuIC) analysis (Saijo et al., 2017) or tau biosensor cells (Holmes et al., 2014; Takeda et al., 2016) supports this tau spreading hypothesis.

In addition to being trans-cellularly propagated, tau may have specific functions in the extracellular space. For example, the application of extracellular tau increases the electrical activity of iPSC-derived or primary cortical neurons (Bright et al., 2015). Moreover, extracellular tau may elicit cellular toxicity under certain conditions. The addition of tau into the cell culture media binds to muscarinic acetylcholine receptors and increases intracellular calcium, eventually leading to cell death (Gómez-Ramos et al., 2006, 2008). Furthermore, extracellular oligomeric tau impairs memory and long-term potentiation (LTP) in mice (Fá et al., 2016; Puzzo et al., 2017). However, in some experimental models, the amount of tau needed to elicit impairment is far more than the circulating levels of tau; thus, further investigation is needed to determine whether aforementioned effects indeed reflect the pathophysiological function of extracellular tau.

## Possible mechanisms of tau release

The amount of soluble monomeric tau released from cells is altered by the various types of external stimuli. For example, starvation or lysosomal dysfunction increases tau release (Mohamed et al., 2014). In addition, stimulating neuronal activity in either cultured neurons or *in vivo* also enhances tau release (Pooler et al., 2013; Yamada et al., 2014; Fá et al., 2016). Given that extracellular tau influences neuronal activity (Bright et al., 2015), this observation suggests that the activity-dependent release of tau participates in a positive feedback loop on neuronal activity.

Differences in tau species or isoforms also impact its release. For example, extracellular tau levels are differentially altered by the presence of mutations in *MAPT*, an amino-terminal insertion sequence, or a repeat domain (Karch et al., 2012). Moreover, hyperphosphorylation-mimicking mutant tau is preferentially released while extracellular tau is rather less phosphorylated compared to intracellular tau (Plouffe et al., 2012; Bright et al., 2015). Furthermore, caspase-3 cleavage at D421 increases tau release (Plouffe et al., 2012). These observations of variable tau release suggest the presence of active cellular mechanisms regulating its release.

Tau does not contain an apparent signal peptide sequence to regulate its trans-location to the endoplasmic reticulum (ER) for conventional secretory pathway, thus, it remains to be elucidated how tau reaches the extracellular space. In addition to tau, an increasing number of proteins without a signal peptide sequence are being recognized for their unexpected release into the extracellular space. Such secretion is termed unconventional protein secretion and occurs via vesicular or non-vesicular pathways.

One of the best-characterized mechanisms for unconventional vesicular secretion is the exosome-dependent pathway. Exosomes are the extracellular vesicles that are released upon fusion of the multivesicular bodies with the plasma membrane. Tau from the CSF (Saman et al., 2012) and blood of patients with AD (Fiandaca et al., 2015) is associated with exosomes. N2a cells over-expressing tau (Wang et al., 2017) and microglia also release tau in an exosome-dependent manner (Asai et al., 2015). However, other studies show that the majority of extracellular tau is membrane-free (Chai et al., 2012; Karch et al., 2012) and not associated with extracellular vesicles such as exosomes or ectosomes (i.e., plasma-membrane originating vesicles) (Dujardin et al., 2014). Therefore, the exosome-dependent mechanism may only explain a small fraction of the total tau that is released into the extracellular space.

Current understanding of exosome-independent tau release mechanisms is limited, however, chaperones and proteins responsible for intracellular vesicle trafficking have been implicated. For example, DnaJ/Hsc70 chaperone complexes control the release of tau and knockdown of SNAP23, a SNARE protein involved in vesicle-mediated exocytosis, blocks tau release (Fontaine et al., 2016). Interestingly, a significant portion of endogenous tau in neurons is associated with membrane fractions and membrane-associated tau is largely dephosphorylated, like extracellular tau (Pooler et al., 2012). Although the identity of membrane-associated tau remains to be clarified, it is tempting to speculate that tau is translocated into intracellular vesicles prior to its release.

Rab GTPase regulates various steps of intracellular vesicle trafficking and membrane fusion. Rab7a (Rodriguez et al., 2017) and Rab1a (Mohamed et al., 2017) have been recently implicated in tau release, further suggesting that tau release involves vesicle transport. Elucidating the precise molecular mechanism involving these proteins will uncover release pathway in more detail.

Some substrates such as fibroblast growth factor 2 are secreted via the unconventional secretion pathway by direct translocation across the plasma membrane (La Venuta et al., 2015). Tau also interacts with plasma membrane via amino terminal projection domain (Brandt et al., 1995), which is influenced by a tauopathy-associated mutation (Gauthier-Kemper et al., 2011). Nevertheless, there is no evidence to date that tau is also released through such a non-vesicular secretion pathway.

The majority of tau binds to microtubules (Weissmann et al., 2009), thus one might speculate that detachment of tau from microtubules increase releasable pool of tau. However, the relationship between tau release and interaction with microtubules is not understood well. To date, there is no clear demonstration that microtubule binding properties directly influence tau release. The stabilization or destabilization of microtubules with paclitaxel or pseudolaric acid B both increased tau release (Karch et al., 2012). This may be the consequence of altered homeostasis of tau by perturbation of microtubule binding. How the release of tau is related to its dynamic kiss-and-hop interaction with microtubules needs further investigation.

Finally, the majority of current literature only describes the release of soluble monomeric tau and there is little definitive evidence of whether and how seed-competent tau is released into the extracellular space. Soluble monomeric tau and seed-competent tau may exit cells via distinct pathways. Because tau aggregates that leak from dying or degenerating neurons also likely mediate transcellular propagation, further studies are needed to investigate whether there are active release mechanisms for seed-competent tau from living cells.

## Clearance of tau from the extracellular space

Extracellular tau levels are maintained based on a balance between cellular release and clearance; thus, changes in clearance will also affect extracellular tau levels. A study of regulatable tau transgenic mice found a relatively low turnover rate of ISF tau (the expected half-life was ~11–17 days) (Yamada et al., 2015); nonetheless, the molecular mechanism for clearing extracellular tau remains largely uninvestigated.

The paravascular clearance pathway, also termed the “glymphatic system,” has been recently recognized for its critical role in eliminating extracellular molecules from the brain via perivascular tunnels (Iliff et al., 2012). This pathway may participate in tau clearance, as tau accumulates along the veins upon injection into the brain (Iliff et al., 2014).

Uptake and degradation of seed-competent tau by glial cells may inhibit propagation by blocking neuronal uptake. Anti-tau antibodies facilitate microglial uptake of tau aggregates (Funk et al., 2015; Luo et al., 2015; Yanamandra et al., 2015). In addition to microglia-mediated degradation, a brain-to-blood clearance pathway may play a role. A study has shown that tau injected into the CSF appeared in plasma and underwent degradation (Yanamandra et al., 2017). Although the precise mechanism remains to be elucidated, these data suggest the presence of pathway that eliminates extracellular tau from the CNS to the peripheral bloodstream.

## Conclusion

Accumulating evidence suggests that tau is physiologically released into the extracellular space independently of cell death or neurodegeneration. This evidence suggests the novel possibility that tau may play pivotal extracellular roles. Given that tau propagation is mediated by extracellular seed-competent tau, a possible therapeutic intervention strategy is to reduce such pathological tau levels in the extracellular space. However, such a strategy may interfere with the yet-to-be-identified physiological functions of normal extracellular tau.

Elevated CSF tau is a diagnostic biomarker in AD patients (Tapiola et al., 2009). The physiological presence of extracellular tau may challenge the view that this elevation merely reflects the degree of neurodegeneration. Notably, CSF tau levels begin to increase during the period when a significant neuronal loss would not be expected (Bateman et al., 2012). Consistent with this observation, CSF tau in *APP* transgenic mice is increased in an age-dependent manner without a global neuronal loss (Maia et al., 2013).

Elucidating the precise mechanisms regulating extracellular tau levels, especially mechanisms involving prior to neurodegeneration, will lead to important new insights to better understand the physiological/pathological roles of tau in the brain and improve the diagnosis and treatment of tauopathies.

## Author contributions

The author confirms being the sole contributor of this work and approved it for publication.

### Conflict of interest statement

The author declares that the research was conducted in the absence of any commercial or financial relationships that could be construed as a potential conflict of interest.
